# A Culture-Centered Approach to Experiences of the Coronavirus Pandemic Lockdown Among Internal Migrants in India

**DOI:** 10.1177/00027642211000392

**Published:** 2021-09

**Authors:** Devalina Mookerjee, Sujoy Chakravarty, Shubhabrata Roy, Anirudh Tagat, Shagata Mukherjee

**Affiliations:** 1 Jadavpur University, Kolkata, West Bengal, India; 2 Jawaharlal Nehru University, New Delhi, India; 3 Behavioural Insights, Architecture, and Strategy (BIAS), New Delhi, India; 4 Monk Prayogshala, Mumbai, India; 5 Meghnad Desai Academy of Economics, Mumbai, India

**Keywords:** India, migrant worker, reverse migration, health of migrants, CCA, economic problems of migrants, stigma, agency

## Abstract

India’s coronavirus lockdown forced low-wage migrant workers to return from the city to the home towns and villages from which they came. Pre-pandemic living and working conditions were already stressful and difficult for these migrants. The lockdown became an additional burden, since it shut down sources of income with no assurance about when, or if, work and earning to support families could be resumed. This article draws on the lens of the Culture-Centered Approach (CCA) to understand how workers engaged with and navigated these difficult times. A total of 54 migrant workers locked-down at home across the Indian states of Bihar, Uttar Pradesh, and West Bengal were interviewed for this qualitative study. Financial worries were found to be endemic, with rising debt a major source of stress, and educational qualifications becoming an obstacle to earning. Returning migrants were suspected of bringing the virus from the city, and so stigmatized in their home towns and villages. However, the pandemic lockdown also showed some unexpected healthful consequences. It provided these marginalized, and always busy workers the time and space to stop working for a while, to stay home, eat home food, and take walks in the comparatively green and clean spaces of their home environments. In this, the pandemic lockdown may be seen to have enabled a measure of agency and health in the lives of these workers, an oasis albeit temporary, and ultimately subject to the demands of the globalized cities of India.

In India, internal migration sees a large movement of workers from less urbanized spaces to more urbanized ones (Bhagat & Keshri, 2018). Srivastava (2020) points out that India’s largely rural to urban internal migrants comprise “the most vulnerable section of the working poor” (p. 3). Dandekar and Ghai (2020) dismiss the notion of opportunity-oriented migration among this marginalized population. Pointing to the low socioeconomic status of most source areas for internal migrants, they say that this migration “is for subsistence and survival, and falls under the citatory of distress migration” (p. 29). If difficult conditions at home propel the working poor to move to cities, the conditions there are not much easier. Internal migrants to urban India work largely in the construction, manufacturing, and service sectors of the informal economy, where they are exploited by employers or middlemen, and frequently denied basic rights such as adequate wages, nutrition, housing, sanitation and health care (Chatterjee, 2006).

To a population already marginalized in their everyday experience, the pandemic and lockdown came as an additional, unexpected burden. In a document called “Psychosocial issues among migrants during COVID19,” Government of India, Ministry of Health and Family Welfare (2020b) listed the difficulties faced by workers in the pandemic. Issued in April, when workers were reverse-migrating home to villages and small towns, the list states that poor migrant labor are among the most marginalized in Indian society, and that

(C)oncerns faced by such migrant workers relate to food, shelter, healthcare, fear of getting infected or spreading the infection, loss of wages, concerns about the family, anxiety and fear. Sometimes they also face harassment and negative reactions from the local community” (p. 1).

These apprehensions were made concrete by media reports that showed state-provided trains and buses overwhelmed by workers wanting to go back home. Cameras at the outskirts of Indian cities recorded an exodus of the migrant poor, headed back to the safety of home towns and villages. Infection had become a real fear in cities, and lockdown had closed down sources of income, so a return to the places from which they came was the only option for migrants (Guha et al., 2020).

Those who had a bicycle started to ride (Sengupta & Mohanty, 2020), those who did not get, or could not afford transport started walking under the Indian summer sun (Pal & Siddiqui, 2020; Press Trust of India, 2020a). Trains, buses, and workers with their families on foot got stranded at, or rerouted from state borders as lockdown restrictions set in across states (Jebaraj, 2020). With food and water in short supply while workers travelled (Bhagat et al., 2020), India’s coronavirus reverse migration killed at least 200 migrants over 2 months (Banerji, 2020), and rapidly became what Biswas (2020) called a “human tragedy.”

On May 12, the Prime Minister addressed the nation (Prime Minister’s Office, India, 2020). In a speech that millions heard live at this dire time, his speech did not lay down any concrete measures to help those in distress during the pandemic lockdown. Instead, it delineated a vision of a future cast in “Hindu economics,” in which Indians were to be more self-reliant, and responsible for their own futures (Sagar, 2020). On May 23, the Ministry of Home Affairs announced that 7.5 million migrant workers had returned to their hometowns and villages (Press Trust of India, 2020b). Once back, migrants served the mandated quarantine in local state-organized facilities (Government of India, Ministry of Health and Family Welfare, 2020a). This done, they were free to return to their families and homes.

Now located at home, reverse migrant workers began the long wait through the pandemic lockdown. It is important to understand the experiences of workers in this period of limbo because a large number of reverse migrants did not have income through this time, and were back home with little understanding of what the future may hold (Guha et al., 2020). Added to this stress was the need for behavior change (mask, sanitize, social distance), to minimize the possibility of getting, or passing on the virus. A loss of income, a change of living conditions, and behavior changes that need constant mindfulness are large-scale changes in people’s lived experience, all considered likely to cause significant stress (Scully et al., 2000).

## Literature Review

Low-wage migration for work is a difficult and risky experience worldwide (Sweileh, 2018). Existing at the margins of host cultures, migrants do the most difficult, low-paid, and dangerous work, with access to the least safety or insurance (Ahonen et al., 2007; Moyce & Schenker, 2018). Migrants are vulnerable to psychosocial conditions such as stress and depression around issues of work, earning, life-choices and identity (Adhikary et al., 2011; Lee et al., 2012; Sun & Dutta, 2016), social competence (Wong & Chang, 2010), as well as injuries and other occupation-related physical conditions (Hargreaves et al., 2019).

### Internal Migration and Health Outcomes in the Pandemic

India’s working-class internal migrants echo this larger pattern. Virupaksha et al. (2014) point out that migration brings with it lifestyle and self-esteem issues. Bhardwaj et al. (2012) saw anxieties about social support, relationship problems, worries about health and stressful emotional reactions, and a lack of satisfaction with living conditions. In a review of the literature on health and health care of internal migrants, Kusuma and Babu (2018) found that they suffer widely from noncommunicable conditions such as diabetes and hypertension, as well as communicable ones such as HIV. Migrants are frequent sufferers of malaria. They also experience multiple barriers to health care access.

With reference to the possible long-term consequences of the pandemic lockdown on the health of migrants, Andrade (2020) comments that the risks of mental illness such as depression or other psychosomatic stress disorders may last some time, because of the possibility of an “imprinting of vulnerability into neuronal circuits” (p. 247). Choudhari (2020) lists the vulnerabilities of internal migrant workers during the lockdown. This comprehensive list includes susceptibility to communicable diseases, absence of support, depression, stress, social exclusion, economic constraints due to loss of work, issues with following the rules and regulations of personal safety, and peritraumatic psychological distress during the pandemic. It is not an exaggeration to say that Andrade’s (2020) and Choudhari’s (2020) assessments point to the possibility of a substantial public health crisis in the foreseeable future.

Not enough is known about the kinds of stressors returning migrant workers have experienced at hometowns and villages, with loss of income, the hazard of infection, and an uncertain future. The central government had announced limited financial support to migrants (Mishra, 2020), systems for quarantine, testing and treatment, and food provisions packages (Bhagat et al., 2020). But time constraints and the scale of the migration meant that these measures were planned in an emergency top–down manner, and implemented in haste (Mohan, 2020).

This study aims to understand the experiences of migrant workers, toward who such top–down policy making is directed. It approaches this aim through listening to migrant’s voices as they tell of the experiences of living through, and dealing with the pandemic lockdown. The research question for this study is, what are internal migrants’ perspectives of the constraining and enabling experiences they are living through and dealing with, during the period of pandemic lockdown? The long-term objective is to be able to suggest entry points toward change in future policy and planning that are grounded in the voiced needs of vulnerable migrants (Dutta, 2008).

## The Culture-Centered Approach (CCA): Culture, Structure, and Agency

The CCA is “a metatheoretical framework that critiques the imbalances of power, uncovering how structures suppress the agency of those in the margins” (Kaur-Gill & Dutta, 2020, p. 133). In this framework, the nuanced interplay between culture, structure and agency becomes a lens to explore and amplify the voices of marginalized subaltern people, who have been silenced by mainstream discourses and practices. Listening to the voice of the subaltern breaks the mainstream silence imposed on subaltern lives, and is both grounded in, and a route to transformative politics (Dutta & Kaur-Gill, 2018). The CCA is an appropriate lens to use here because this study focuses on the voices of marginalized internal migrant workers who have been “systematically erased from the dominant discursive spaces of knowledge production” (Dutta, 2011, p. 3), and are considered of value only as voiceless laboring bodies in the neoliberal cities of India (Kaur-Gill & Dutta, 2020). These workers have experienced three levels of marginalization and being rendered invisible—first, as internal migrants; second, as those affected by the pandemic lockdown; and third, as of no use to the neoliberal state during this time because there is little work available. This is why it is so important to record and understand these voices, at this time, and to use a lens that combines culture, structure, and agency; in order to try and render these invisibilities, visible.

In the CCA, culture is “a complex web of shared practices, beliefs, and values that offers an anchor for interpretive frames” (Dutta, 2017, p. 3). Local meanings draw on cultural understandings that may be understood against, and with, the contexts in which they take place, and cultural contexts are dynamic, constitutive, and transformative in the area of health meanings (Dutta & Jamil, 2013). Structure and agency make up the two other parts of the culture, structure, agency triad (Dutta, 2017). Structure is made of “the material realities that constrain and enable human action” (p. 3), which includes systems and institutions within which people live or with which they meaningfully interact; and agency is the ability of people to navigate, negotiate, or otherwise deal with and work around the constraining factors in their environments, while allowing the enabling factors to help them. This triad, which aims to locate the agency of people within the framework of local cultural contexts and structures, is called the culture-centered approach (Dutta, 2008).

In a CCA study among migrant Bangladeshi construction labor in Singapore, Dutta (2017) found that when articulations were turned away from the dominant discourse of efficiency and effective management and toward the workers themselves, the other discourse that emerged had to do with health safety, and meaningful mechanisms for redressal of their issues. Using social class as a framework for precarious work, Kaur-Gill and Dutta (2020) found that listening to the voices of female domestic workers in Noida (India) and Singapore revealed the many inversions in Asia’s shining cities that disrupt the dominant discourses of mobility and wealth that undergird these spaces. Dutta and Jamil (2013) saw that among low-income Bangladeshi immigrants in New York City, health is both an individual, and a collective construct. This simultaneity contradicts the ideas in cross-cultural communication that classify cultures as either individual or collective.

Following Dutta (2017), and adapting to the context of reverse-migrating workers in this study, culture here includes the practices, values, and beliefs of worker’s home environments, as well as their understandings of being migrant workers with exposure to worlds beyond hometowns and villages. Structure here includes the economic systems and other infrastructural constraints to which they are subject, and agency is the coping behaviors and understandings evolved by those experiencing the pandemic lockdown in their cultural contexts, and subject to the structures in these contexts.

Migrant workers have had to experience and navigate the enormous challenges of return migration during pandemic, and its subsequent state of limbo. By foregrounding triple-marginalized voices in subaltern spaces, trying to understand the role of structural constraints in cultural context, and attending to agency in the subaltern’s navigation of the world (Dutta, 2008; Kaur-Gill & Dutta, 2020), this study aims to disrupt the invisibilities that have shrouded migrant experiences of health, work, and home, in the situational context of the months of pandemic lockdown.

## Method

The primary source of data for this research is semistructured, in-depth interviews with internal migrants in India, who have reverse-migrated to their home towns and villages in the states of Bihar, Uttar Pradesh (UP), and West Bengal (WB). These states are significant sources for internal outmigration in the country (Bhagat et al., 2020). Interviews were conducted about 3 months into the lockdown, when the national lockdown of transport made face-to-face interactions impossible. The consequent lack of data in visual observations and images is the first limitation of this study.

### Sample

The sampling for this study is purposive, with 18 participants from each of the three states making a total of 54. Sampling took into account religion, education, and the latest period as outmigrant.

Religion was used in the sampling because India’s secular fabric is under stress, with severe marginalization of the Muslim minority (Subramanian, 2020). The 80% Hindu majority vastly outnumbers India’s 15% Muslims (Maizland, 2020). In the absence of current data about the religions of internal migrants, the sample had 36 Hindus and 18 Muslims, in a very rough reflection of the disparity in population.

Education was included in the sampling because it is an important factor in access to work across urban and rural India. While formal education does not guarantee income, lower grades of education usually restrict opportunities to the informal sector (Kumar & Sahu, 2013). In order to see if differences in education, and therefore access to work, could show differences in lockdown experiences, the sample included 21 workers with education below the 10th grade, 21 workers with education between the 10th and 12th grades, and 12 graduates.

Period as outmigrant was used to include different patterns of migration. The range of work migration is broad in India, running from relatively stable contract employment, to seasonal agriculture daily wage labor (Srivastava, 2012). The period of more or less than 6 months is used to differentiate between circular patterns of migration for work by the National Sample Survey. Accordingly, the sample for this study included 21 participants who had been migrants for less than 6 months, and 33 participants who had been migrants for over 6 months.

### Recruitment

Participants were recruited purposively from within communities during the lockdown. Local recruiters known to one of the authors through previous projects, introduced the study to prospective participants, got contact details from those willing to participate, and later assisted participants to complete consent forms before interviews. The research protocol received approval from the Monk Prayogshala Institutional Review Board, which is complaint under the U.S. Department of Health and Human Services, and specializes in ethical review for social science studies in India. Informed consent was sought from all participants in line with APA ethical guidelines.

It must be noted that the sample does not include any women. This is because recruitment during lockdown did not find women reverse migrants willing to take time out of their busy days to talk to a researcher. This is the second limitation of the study.

### Data Gathering

The semistructured interview guide asked participants how the pandemic and lockdown had effected their lives. Drawing on Choudhari’s (2020) list of migrant vulnerabilities in the pandemic lockdown, it included open-ended questions about experiences during this time, in the areas of work and earning (Have you had income through the lockdown? Have your earnings been enough for your family expenses?); social relationships (What effect has the corona lockdown had on your relationships with family? Neighbors? Others in the community?); infection (Did anyone you know get infected?), and safety measures (What do you do to try and keep safe at this time?).

Interviews were conducted on the phone by researchers from Delhi. The voluntary nature of participation was emphasized at the beginning of interviews, and participants reminded that they could exit anytime. Interviews were conducted in Hindi or Bengali according to the comfort of the participant, and lasted 30 to 40 minutes each. They were recorded on in-phone apps, translated, and transcribed by the researchers conducting the interviews. In addition, researchers noted their observations and understandings of the in-phone interactions, and these were included in the data. Identity information and interviews were stored securely, with only the researchers in the study allowed access. Names have been changed to protect the anonymity of participants.

### Analysis

The analysis of the data followed open, focused, and axial coding as described in Charmaz (2006). The data were open-coded by the first author to identify emerging themes. This process began after the first interview in each state was conducted, and continued in a process of constant comparison (Glaser & Strauss, 1967). A total of 62 codes were evolved through the process of open coding. Lack of earning, mounting debt, unsafeness of city life, the feeling of home, and worries about infection were among the codes strong enough warrant selective coding. This process yielded the core categories, which included home, city, money, and infection.

From the 62 open codes, the authors evolved 19 focused codes, among which were, making a living; coronavirus fears; home; city as migrant; safety in pandemic, coping understandings, and coping behavior. Each code was at the center of clusters of different open-coded experiences and perspectives about it. In the next process of axial coding, codes and their clusters were examined for their relationships with other codes and clusters. The themes and understandings that emerged from the process of analysis are discussed below.

## Findings

The findings from this study reflect the stressors experienced by male internal migrant workers who have returned home during the pandemic lockdown. In this section, we focus on the health concerns of these workers around two broad dimensions: stress about earning, debt, and work; and coronavirus fears and stigma in the context of home.

### The Stresses of Economic Limbo

Migrants experienced multiple economic stressors during the pandemic lockdown, including the loss of savings and growing debt, anxieties about work and earning at home, and uncertainties about earning in the future.

#### The Health Ramifications of Economic Decline

Most participants in the study were the principal earners of their households. Loss of income and mounting debt has led to serious economic stress for these migrants. As Anwar (36, WB) put it, “If I don’t earn, who will feed my family? My wife does not keep well, who will pay for her treatment?” The themes of income required for food and health care were echoed across interviews across states. It is important to note that economic stress due to pandemic lockdown was reported in every one of the 54 interviews conducted in this study. Om Prakash (40, Bihar) worried,

I had savings that lasted the first two months, I thought that would be enough. But now it’s been three months. We are somehow going on. When I came back (from the city) I thought, after all, how long could lockdown go on? Everybody thought we would be back at work by June-July. But this is just going on [ . . . ] I had to borrow from my brother-in-law, he has some work even now. But how much can I borrow from him? He has a family to run too.

Having run out of savings, or being close to running out of savings were recurrent ideas in the interviews. Participants reported borrowing from extended family, members of the community with greater means, or local money-lenders at rates of interest that they did not reveal. This is worrisome, given that debt-traps can ensure that a household stays in debt for a long time (Karlan et al., 2019).

Loans taken from family or community sources come with another dimension of worry. Participants said they strain relationships when loans cannot be paid back, or a further loan is needed without the means to repay the initial loan. Anish (40, Bihar) worried,

I borrowed 3,000 Rs. from my cousin, but that ran out. Still, I could not find work, so I borrowed again, from a local businessman who was my friend. Now I will need to borrow again, but I don’t know who to ask, I don’t know anyone else who can lend money. No one has extra money now [ . . . ] I know they are annoyed that I did not pay back.

Anxiety about mounting debt was a recurrent theme in the interviews, along with sense of doors closing within communities for repeat borrowers. Participants did not know when they would be in a position to pay back the loans they had taken. Rajnish (39, UP) feared, “I don’t know when I can go back to work there (in the city), how will I pay back?” Bibek (30, WB) explained what he said was his biggest source of stress:

I was gone for only 4 months (as a migrant), it was good money, but only 4 months. I had very little savings when I came back, but my family is here, so it was all right in the beginning. But my child fell ill, so I took a loan for the medicines, a small one. When that money finished and I took another one, a little bigger one. [ . . . ] I worry all the time about it, it is a constant tension in my mind.

The stress of this kind of constant anxiety is cumulative, and holds the risk of numerous health complications of the nervous and immunity systems (Kemeny, 2003). Participants were aware that “tension” is bad for health, but those trying to assert their agency in this adverse situation by trying to find work were met with other structural constraints, as discussed in the next section.

#### Work and Earning at Home

Participants reported that while opportunities to earn were available in hometowns and villages, such opportunities were limited to agriculture and construction, contracted day-to-day, and offered lower pay than they were used to for comparable work in the city. Shahnawaz (36, UP) described the process of getting this work:

I go and wait in one place in the village, and the truck comes and picks us up to take us to the work-spot. We work the whole day, they pay very little for a day. Sometimes there will be work for 5 days, or a week, but that’s all. [ . . . ] Not everyone who waits gets work for the day. I go every day to wait at that place where they pick up.

Shahnawaz and other migrants said that there is less work available in hometowns and villages than in the city. So, while some migrants with backgrounds of manual labor reported occasionally finding work, many did not, and those overqualified for available work found no earnings. Anil (31, Bihar) who has a BA degree, sorrowed that education had become a constraint to him:

Here there is only building work, or work on the field [agricultural], my BA is of no use here. I have never done laborer work like this. In the city I do work that needs reading and writing, here there is no work like that. I cannot earn until I go back to the city. [ . . . ] But how will I feed my family till then?

This inversion of the role of education in the context of pandemic was echoed by others overqualified for the work available at home. “What’s the point of my education?” said Sonjoy (WB, 27) bitterly, “if it is not of use to feed my family?”

Structural support from the state in terms of continuing access to food and other basics could be enablers with the power to alleviate this distress, but this research found that such support had been uneven. Some participants in WB reported receiving partial support of edible grain and other foodstuff from the state. Those from Bihar who had received this kind of support said that the foodstuff they received had worms, was rotten or stale enough not to be edible. Not a single participant in UP had received food or any other kind of support from the state through this time.

A related theme running through the interviews was projections of a future return to normal, in which workers could return to work in the city. A postpandemic normal is defined as one in which work and earning would be possible again, migrants could return safely to the city to resume earning, and remit money back home. Vijay (43, UP) said,

I work to feed my family, give my children here [at home] a future. I thought I would also earn enough this year [as a migrant] to repair the house. Now I am sitting at home and waiting for months, nothing to do, no work. Only when I can go back to work, send money home, will I feel that corona is over.

The future resumption of responsibilities that have remained unfulfilled through the pandemic marks both something to look forward to, and an inevitable end to the break from the drudgery of migrant work. The interviews indicate that workers like being home, and are fearful that they may have to return to the city before a vaccine or cure is found. But they are aware that the structural factor of economic contingency is a more important factor in their decision making than their safety-related preferences. As Bilal (30, UP) said, “This person says cure, that person says vaccine, but who will feed us?”

The CCA points out that the structural constraints within which people live have material and psychosocial consequences on their health (Dutta, 2017). With incomes and remittances at a halt over the pandemic lockdown, no formal systems with adequate coverage for loans or food security, and no end to the pandemic or lockdown in sight, the precarity of existing within the multiple disenabling structures (Kaur-Gill & Dutta, 2020) that surround reverse migrants have become sources of serious stress, and obstacles to their health.

### Home as Health, and the Stigma of a Return in the Context of Pandemic

Even while economic stress plagues the experience of return migrants, they feel that being home is associated with positive health practices and impacts. Unfortunately, however, these positive effects are moderated by the stigma they have experienced as people returning from the city in the context of infectious pandemic.

#### Healthier at Home Than in the City

The participants in the study saw health in the context of pandemic as related to food, open air, care, and time. Nadeem, (31, Bihar) said, “if you have good food, go out and walk for a while quietly in the morning or evening in the open air, that’s all you need to stay healthy.” Bijoy (40, WB) echoed this sentiment, “I put on my mask and walk for a while every day, there is plenty of space [ . . . ] I have time to do that. The food is good, home-cooked, I eat well. Everything tastes better here.”

Food cooked at home by a loved one’s “own hands” is an important dimension of good food. As Manohar (32, UP) put it,

(M)y mother cooks for everyone with her own hands, she has decided that we should eat more vegetables, dal, healthy food for COVID. So we eat lots of good food, she cooks different things, we sit, she sits with us, and we eat.

These aspects of good, healthy food—care, variety, and the time to sit and eat, are missing in the city. Eating in the city is a rushed, unsatisfactory experience, with men cooking for themselves. Mukhtarul, (38, WB) said,

There are eight of us living in a room, we have different times of work, always coming and going. One person cooks, and everyone eats whenever they can. Most days I eat cold food, or the person has cooked in a hurry and left to work, there’s no taste.

Just as the city is missing the essential dimensions that contribute to healthy food, it is also missing another component of a healthy life, which is space to breathe made of open, clean air. Jeet (29, Bihar) was eloquent about this difference:

There (city) there is no place to breathe, it is hard to breathe. You can see a thick layer of dirt on everything. You can’t get away from the dirt and the pollution in the air. But here, I go for a walk, and there are trees, the air feels clean, I can breathe [ . . . ] My friend said there is more oxygen here, I don’t know all that, but here it’s no problem to even run with my mask on.

In the cities at which migrants work, health connotes the things it is good to stay away from—pollution, bad food, difficult living, and working conditions. At home, however, health is something they can reach for, with home-cooked food, open areas and time for exercise and relaxation, and the comfort of being surrounded by those who care.

Another aspect of home that promotes safety during pandemic pertains to space. Participants said that social distance was very difficult to maintain through the living and working conditions of migration. Satendar (36, UP), commented,

This six feet distance, it is not possible in the city. All day, someone or the other is so close that you can touch. Sometimes they push when they pass by, they pass so fast you can’t even tell them to stay away. Even vehicles, motorcycles, cars, they come so close.

In contrast, village or small town spaces are reported to less populated, and open. “I don’t usually meet even one person when I go to the fields in the morning,” said Ravidas, (30, Bihar) “so there’s no need to wear a mask all the time during that walk.”

The feeling that home is a bastion of safety finds reflection in other experiences of participants. Masks were reportedly not worn at home across states, nor hands washed regularly when home. This is because homes, and families, are perceived to be no-risk zones. As Shahid (34, UP) put it,

I go out only when needed. At home there’s my parents, younger brother, my wife and two children. Sometimes my brother will go to the nearby market, but he bathes with soap when he comes back, everything is washed ( . . . ) My parents are aged and stay at home, my wife stays at home too, and the children have been at home for two months now.

Participants said that they did not consider it possible to get infected from their families either because no other member of the family left the house during the lockdown, or because any family member going out only went to spaces within the locality, and followed safety directives (mask, hand cleanliness, social distance, etc.). Being close to home and loved ones was essential to these participants in keeping their health safe during the pandemic. Care, time, open air, and good food are all important to this process, and these are only available at home.

The reluctance to return to the city was a recurrent theme in the interviews. Hamid (42, Bihar) said that if there was a choice, he would not return at all.

Who wants to go back there? I would say here if there was work. Then I would relax with my family, eat like this every day! I will stay as long as I can [ . . . ] But I will have to go back there, we all know that.

Others echoed this idea, and said that they would extend their stay at home as long as they could. But these voices are united in their belief that sooner or later, they will have to return to the city.

In the larger context of the neoliberal state which continuously calls on the laboring bodies of workers, staying home is a subversive act of profound agency. The escape home, and the refusal to leave home until structural constraints are so overwhelming that there is no choice but to find a way to start earning again, these are assertions of agency in the face of the compulsions of laboring in the neoliberal world, as it is defined in the CCA (Dutta, 2008). Such acts of agency have enabled migrant workers to access what they need for a healthy life. This is a small space in which to maneuver, bracketed by the triple-marginalizing contingencies of the pandemic lockdown. But migrants are keenly aware of the gift of this time at home with family, in spite of all the other troubles that surround them.

Dutta and Jamil (2013) saw that among Bangladeshi immigrants, “The personal ownership of health is interconnected with the relational, familial, and collective ownership of health” (p. 175). Here too, the care of family and access to community resources such as open land are identified by migrants as elements that bring and keep good health in their lives. These acts of subversion (in the context of the larger drudgeries of migrant life) may be seen as having given them a space and time to, however temporarily, reclaim agency in their own health.

#### Returning Home to Stigma

Hometowns and villages are an invaluable refuge at this time. But the return home during pandemic has also created a kind of marginalization for migrants within this refuge, by marking them as possible carriers of infection. Mangal (33, WB) explained why he felt marked out in this way:

We came all this way, back home. But we couldn’t go straight to our family, we had to stay in the school building [quarantine] for 14 days. Then we went home. [ . . . ] This is a village, everyone knows who is coming back. It’s not like a city, where no one knows anyone. People here say that those who have come back from the city must be infected.

The fear of coronavirus infection is pervasive among both returning migrants, and their communities at home. Migrants feared that they might be carriers themselves, bringing infection to families at home. “After all, I came from the city. Maybe it was on me. Anyone can pass it on [ . . . ] They were safe here before we came,” said Rajnish (39, Bihar). Going through quarantine relieved some of that fear, but also marked migrants as entering the heretofore safe community from spaces of infection.

Participants reported that while families were relieved and pleased to have them back, members of larger communities are very concerned about infection being carried in from the city. Sajid (45, UP) explained,

Everyone knows that corona is worst in the city. Here it is safe, very few cases and not close at all. So they think that people from the city should not be allowed to return. All it takes is one or two people, and the infection will come. That will be very bad, they say.

Participants reported that there has been a hardening of borders of local areas by conservative elements in these areas. While such negative sentiments within communities were thankfully not prominent enough to keep migrants out, the very fact that such an opinion existed was stressful to these migrants.

The consequences of pandemic reverse migrant stigma in small communities are as disconcerting as may be expected, disrupting relationships within the community. “The neighbors on one side stopped talking to my family [ . . . ] we had always had a friendship with them before I came back,” said Roshan (30, UP). Iftekar (43, Bihar) who returned from the city with a seasonal cough and cold, sounded distraught, “Everyone (in his locality) said, he has corona, we will get it, but I did not have corona, I had no illness. They did not believe me.”

The social stigma faced by reverse migrants during the pandemic is an inversion of the usual status of the migrant on his home ground. Migrants are valuable to the economy and society of their home communities in Indian towns and villages. This is due to, first, the economic aspect of earnings and financial remittances which directly and indirectly support family and community back home (Castaldo et al., 2012), and second, because they represent a kind of out-facing view for people in smaller towns and villages, to the larger world and its ways (Bhaskaran, 2011).

The pandemic has challenged this traditionally positive way in which migrants are viewed in their home communities, by turning the mere rumor or possibility of infection into an ostracizable stigma. This marginalization has been deeply upsetting in the experience of those who reported it in the study, because no proof of illness is needed to be stigmatized in this way. Just having returned home from the city in the wake of the pandemic is enough of an offence, to those within communities who want to harden the borders of those spaces, and marginalize already-vulnerable returning migrants.

## Discussion

The period of pandemic lockdown has held several kinds of stressors for return migrants. Financial worries are a constant source of unease, with multiple debts piling up and little sight of a future in which they may be repaid. Debts from family and community have become sources of stress because they negatively affect relationships, and close doors for future borrowing. Dutta (2008) urges researchers to attend to the interplay between culture and structure. Here, a culture of borrowing from known others is transformed into a relational barrier, even as larger structures such as banks, and potential enablers such as government loans, remain unavailable to these marginalized people. The local money-lender fills the gap between family and community lenders, who are reachable, and formal structures like banks, which are not. Although this kind of money-lending is located in the cultural landscape of home, it is also part of an economic structure that causes apprehension, and fear for the future. Any future policy about migrants must prioritize addressing the extreme financial precarity of internal migrants. This study shows the need for a kind of social security or insurance to get workers though low-or-no-income periods, but further research focused on the economic structural constraints experienced by migrant workers is necessary to shape such policy.

Like Dutta and Jamil (2013), this study saw conceptions of health that were both individual and collective—Food cooked by loved ones, time to breathe clean air and walk on common grounds, and enough interpersonal space that infection is not a constant fear, lead to good health. In this conception, meanings of health are co-constructed between people, and also, somewhat extending Dutta and Jamil (2013), between people and the environment. The individual exerts agency to stay at home, and family and community provide positive cultural reasons to do so. In opposition to the healthful environment of home, the dust and lack of trees and open air, and the cold, tasteless food in the city structurally function to constrain health, just as the agentic act to stay home means continuing access to the good food, open space and trees that denote good health to these workers. If the health of workers is to be put at the center of any future agenda of development, then there is a need to find ways to locally create and develop sustainable village and small town economics, so that living at home becomes a real option for these migrant workers.

### The Neoliberal City and Inversions During Lockdown

The analytical lens of the CCA specializes in turning focus away from dominant discourses and toward the voices of those silenced by the workings of those discourses (Dutta, 2008, 2017; Kaur-Gill & Dutta, 2020). The dominant discourse about the pandemic lockdown has been to decry it as a period of low productivity. While this may be true, this study shows that the period also holds some important inversions, which demonstrate the importance of turning away from mainstream discourse to listen to the voice of the subaltern migrant worker.

The neoliberal city may be seen as simultaneously enabling (through work and earning) and constraining the migrant, as it looms as an enormous structural constraint to health. Unpleasant as it is, the city provides resources to support loved ones. The pandemic lockdown has allowed the migrant workers in this study to be home, eat well, and breathe freely. So, the lockdown, which has in many respects been a constraint, has in this particular dimension functioned as an enabler to health.

The pandemic lockdown as an enabler of health is one of three inversions that emerged in this culture-centered study. An inversion is a situation which may reasonably be expected to run one way (pandemic lockdown as a disenabler of health; education as an enabler to find work; the returned migrant as a welcome figure within the community), but in fact runs the other way. The lockdown is found to aid healthy practices; being overqualified for work in the village leads to no income through the lockdown; and return migrants are stigmatized by some within the community as they are perceived to carry the marks of the city, in this case, the infectious coronavirus. These inversions point to the enormous chaos the pandemic lockdown has brought to the lives of migrant participants, and to the multiple layers of constraints they have had to navigate, every day, to survive these experiences with some measure of agency. The CCA regards actions that promote health, even through structural constraints, as acts of agency (Dutta et al., 2017). The migrant worker’s act of leaving the city to return home, his acts of preserving health with walks in the open air, or eating food leisurely and in familial company, and his voiced desire to stay home until conditions improve in the city are all agentic, albeit temporary, and girded from all sides by structural contingencies and constraints.
